# Evaluation of New Zealand's Radiology, Nuclear Medicine, and Medical Imaging Research Output: A Bibliometric‐Based Approach

**DOI:** 10.1002/jmrs.875

**Published:** 2025-02-25

**Authors:** Vicky Li, Sibusiso Mdletshe

**Affiliations:** ^1^ Department of Anatomy and Medical Imaging, Faculty of Medical and Health Sciences The University of Auckland Auckland New Zealand; ^2^ Radiology Department Te Whatu Ora Te Toka Tumai Auckland (Auckland City Hospital) Auckland New Zealand

**Keywords:** Reporting, Research, Review, Writing

## Abstract

**Introduction:**

The use of medical imaging services has increased globally with a concurrent increase in radiology, nuclear medicine and medical imaging (RNMI) research. However, New Zealand's RNMI research output relative to global trends is under‐examined. This project evaluates New Zealand's RNMI research output between 1996 and 2022 compared to selected countries while highlighting global RNMI research output trends.

**Methods:**

A bibliometric‐based performance analysis was conducted using publication data from the SCImago Journal, the Country Rank portal, Clarivate InCites Benchmarking, and the Analytics platform. Registration data of RNMI professionals by country was collected to evaluate the relationship between research output and the number of registered professionals.

**Results:**

Among the seven selected countries (the United States, United Kingdom, Canada, Australia, Ireland, New Zealand and South Africa), New Zealand's research output was low, even when adjusted for population size and the number of professionals. A significant positive correlation was found between the number of registered RNMI professionals and the number of RNMI publications. Despite this, New Zealand had the highest percentage of RNMI documents cited.

**Conclusion:**

Although New Zealand's RNMI publications follow the global upward trend, it does so at a proportionate loss. New Zealand ranked low in most bibliometric indicators apart from the percentage of documents cited, where it showed a notable citation impact. Emphasising research, increasing collaborative efforts, and undertaking further statistical analyses may enhance New Zealand's RNMI research output.

## Graphical abstract

This article presents the results of the evaluation of New Zealand's radiology, nuclear medicine and medical imaging (RNMI) research output between 1996 and 2022 compared to selected countries while highlighting global RNMI research output trends. The results demonstrate that New Zealand's RNMI research output is lower when compared to the selected countries. However, there is a demonstration of growth in recent years.

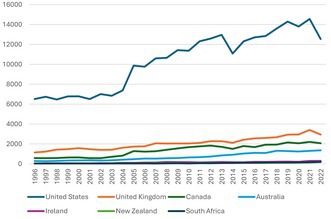

## 1 Introduction

Medical imaging, such as X‐ray imaging, computed tomography, and magnetic resonance imaging, plays an indispensable role in the healthcare system in diagnosing abnormalities, treating diseases and tracking disease progression [1]. The use of medical imaging has significantly increased over the past two decades [2, 3, 4] due to factors like increased demand for services, wider availability of equipment and evolutions in equipment technologies [5]. Studies have highlighted the important role that research has in underpinning these evidence‐based practices, whereby research productivity positively correlates with improved health outcomes and organisational performance [6, 7].

As the use of diagnostic imaging services has increased over the past few decades [3, 4, 8], there has also been a concurrent global increase in the specific subject area of radiology, nuclear medicine, and medical imaging (RNMI) research output [9, 10]. Publication analytics platforms frequently use the RNMI classification to categorise research related to medical imaging sciences. Leading countries for research in the medical imaging sciences include the United States (U.S.), the United Kingdom (U.K.), Australia and Canada [11, 12, 13]. However, there is a gap in the current literature examining New Zealand's RNMI research output and whether it is keeping up with current trends. This is important to gauge New Zealand's contribution to RNMI research, which will also indicate whether the RNMI practice in New Zealand is research‐driven.

This study sought to answer the following question: ‘How does New Zealand's RNMI research output compare to selected countries globally?’. Specifically, this bibliometric study aimed to determine the status of New Zealand's RNMI research output compared to selected countries while highlighting the current trajectory of the RNMI research output globally. The results of this study will be valuable to New Zealand's RNMI research community, encouraging engagement with research and the use of evidence‐based practices in the medical imaging sciences discipline.

## 2 Methods

This study utilised a bibliometric‐based approach, which assesses and quantifies research productivity, quality and impact of publications [14, 15]. It uses statistics to describe trends and highlight relationships between published works [16]. It used existing data from the SCImago Journal and Country Rank (SJCR) portal and the Clarivate InCites Benchmarking and Analytics tool (InCites) to compare the RNMI research output of New Zealand with selected countries between the years 1996 and 2022. The SJCR includes journal and country scientific indicators developed from data within the Scopus database to compare and analyse journals and country rankings [17]. The Clarivate InCites platform is a research analytics tool using the Web of Science Core Collection (WoSCC) as the data source to analyse institutional and individual productivity and impact, compare performance among peers, and monitor collaboration activity [18, 19]. Using two platforms with different bibliometric indicators strengthened the findings and allowed for a comprehensive analysis of the research output. Both platforms consider all document types from their respective databases for analysis.

### 2.1 Research Design

The study used a performance analysis technique, evaluating the contributions of research constituents to a given field [20]. The RNMI research output of New Zealand and other countries was measured through several performance parameters: the total number of published documents, the average number of documents per million residents, the average number of documents per hundred registered professionals, the total number of citations, the total number of citable documents, the total number of self‐citations, the average number of citations per document, the percentage of documents cited, the Category Normalised Citation Impact, the Impact Relative to World, the h‐index, and international and industry collaborations.

A selected number of countries was included in the study, and the following criteria were used to ensure a variety of countries were selected (any of the selected countries had to meet at least one criteria):

- New Zealand's neighbouring countries.
- Countries with approximately equal population sizes to those of New Zealand.
- Countries that are known to be leaders in RNMI research.
- Countries with significant population sizes.
- Developing and developed countries.
- Availability of data regarding the registration of RNMI professionals.

After considering the above criteria, the following countries were selected for inclusion in this study:

- New Zealand (primary research focus).
- Australia (neighbouring country and a leader in RNMI research) [11, 12, 13].
- The United States (significant population size [21] and a leader in RNMI research).
- The United Kingdom (leader in RNMI research).
- Canada (leader in RNMI research).
- Ireland (similar population size to New Zealand).
- South Africa (developing country with a significant population size) [22].

### 2.2 Data Collection

Using the SJCR portal, data was extracted from the subject area of ‘Medicine’ and the subject category of ‘Radiology, Nuclear Medicine and Imaging’ Similarly, on the Clarivate InCites platform, ‘Locations’ was selected as the entity type for analysis, and the research area chosen was ‘Radiology, Nuclear Medicine and Medical Imaging’ These subject areas were chosen as they cover the wide field of medical imaging sciences, aligning with the scope of this study. Selecting the time range of 1996 to 2022 allowed for both a current representation of the research output by country and also accounted for any yearly fluctuations.

The performance parameters used for data extraction from the SJCR and InCites portals are summarised in Table 1, along with their relevance and definitions.

**TABLE 1:** Selected performance parameters extracted from SJCR and InCites to compare the research output of selected countries, including its relevance and definition [20, 23, 24, 25].

Portal	Parameter	Measurement relevance	Definition
SJCR	Total publications	Productivity	Total number of published documents between 1996 and 2022. All document types considered, including citable and non‐citable documents.
	Citable documents	Scientific impact	Total number of citable documents between 1996 and 2022. Includes articles, reviews and conference papers only.
	Citations	Scientific impact	Total number of citations by the documents published between 1996 and 2022.
	Self‐citations	Scientific impact	Total number of self‐citations received by the documents published between 1996 and 2022.
	Citations per document	Scientific impact	Average number of citations per document between 1996 and 2022.
	H‐index	Productivity and impact	Country's number of articles (*h*) that have received at least h citations.
InCites	Total publications	Productivity	Total number of published documents between 1996 and 2022. Includes all document types.
	Times cited	Scientific impact	Number of times the set of publications has been cited between 1996 and 2022.
	% documents cited	Scientific impact	Percentage of publications that have been cited one or more times between 1996 and 2022.
	Category normalised citation impact (CNCI)	Scientific impact (Normalised)	Citation impact (citations per paper) normalised for subject, year, and document type. This indicator shows the impact of the research in a particular subject area. CNCI of 1 = performance at par with the world average. CNCI > 1 = above average. CNCI < 1 = below average. CNCI of 2 = twice the world average.
	Impact relative to world	Scientific impact and relative research performance	Citation impact of the set of publications as a ratio of the world average. This indicator normalises for the year but not the subject. It shows a broader view of the research impact relative to the global research impact, where the world average equals 1. Values exceeding 1 = performing above the world average. Values below 1 = performing below the world average.
	International collaborations	Collaboration	Papers involving one or more international co‐authors.
	Industry collaborations	Collaboration	Papers involving two or more organisations where at least one organisation is listed as corporate or global corporate for its organisation type.

Population data by country from the most recent year, 2022, were extracted from the Population Reference Bureau to normalise the data and factor in New Zealand's smaller population size. Registration data of working RNMI professionals by country were collected from reports between the years 2021 and 2023 by each country's respective professional associations that were publicly available online. This was used to estimate the number of RNMI professionals in each country, mostly involving radiographers, radiation therapists, nuclear medicine technologists and magnetic resonance imaging technologists.

### 2.3 Data Analysis

Data was compiled into Microsoft Excel, and tables and graphs were created as appropriate. Descriptive statistics were used to analyse the data, and inferential statistics using Spearman's rank correlation coefficient was applied to determine the significance (*p* < 0.05) of the data.

### 2.4 Ethical Considerations

The study did not involve human or animal subjects; the data used was collected from the publicly accessible platforms, that is, SJCR, InCites, and selected countries registration data. All the data collected did not have any personal/identifying information.

## 3 Results

**FIGURE 1:**
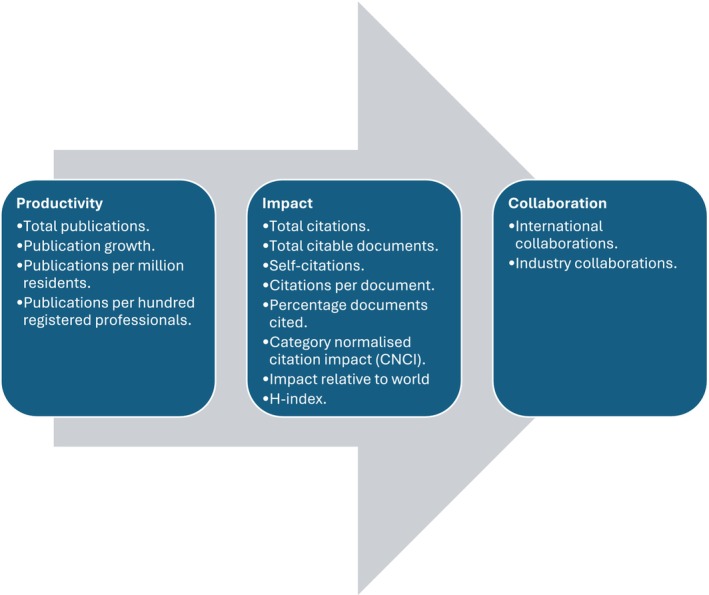
Sequence of results presentation.

The findings are presented as shown in Figure 1, where the productivity indicators are first presented, then the impact indicators and collaboration.

To provide some context, the world population growth was reviewed, and it was found that the total world population grew immensely between the years 1996 and 2021, growing from 5.81 billion to 7.89 billion in 2021 [26]. The review of populations by country in mid‐2022 revealed that New Zealand has a substantially smaller population size of 5.1 million compared to countries like the U.S. (332.8 million) and South Africa (60.6 million) but has a comparable population size to Ireland (5.1 million). Also of note, South Africa is the only country still classified as developing out of the seven selected countries, whereas the other countries are deemed developed [22].

### 3.1 Total Publications

**FIGURE 2:**
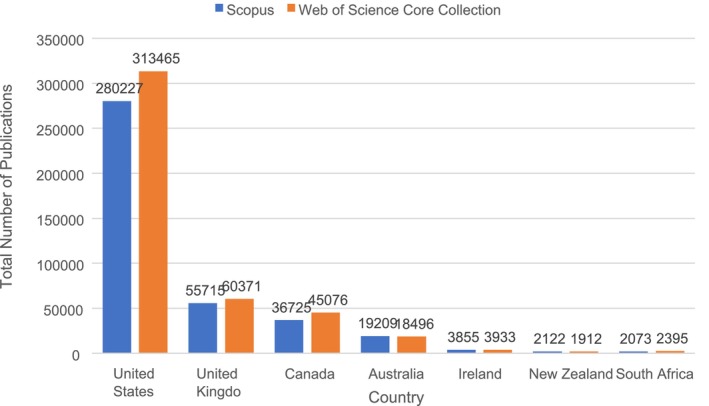
Total number of publications on RNMI between 1996 and 2022 by country on Scopus and WoSCC [27, 28].

Figure 2 shows the total number of RNMI publications for each selected country between 1996 and 2022 on the Scopus and WoSCC databases. The U.S. was found to be in the lead in terms of productivity on both Scopus (280227) and the WoSCC (313465). The U.K. followed the U.S. in second place, Canada in third, Australia in fourth, and Ireland in fifth. According to the Scopus data, New Zealand was placed sixth (2122 publications) and South Africa seventh (2073), whereas the WoSCC ranked South Africa sixth (2395) and New Zealand seventh (1912).

### 3.2 Publication Growth

**FIGURE 3:**
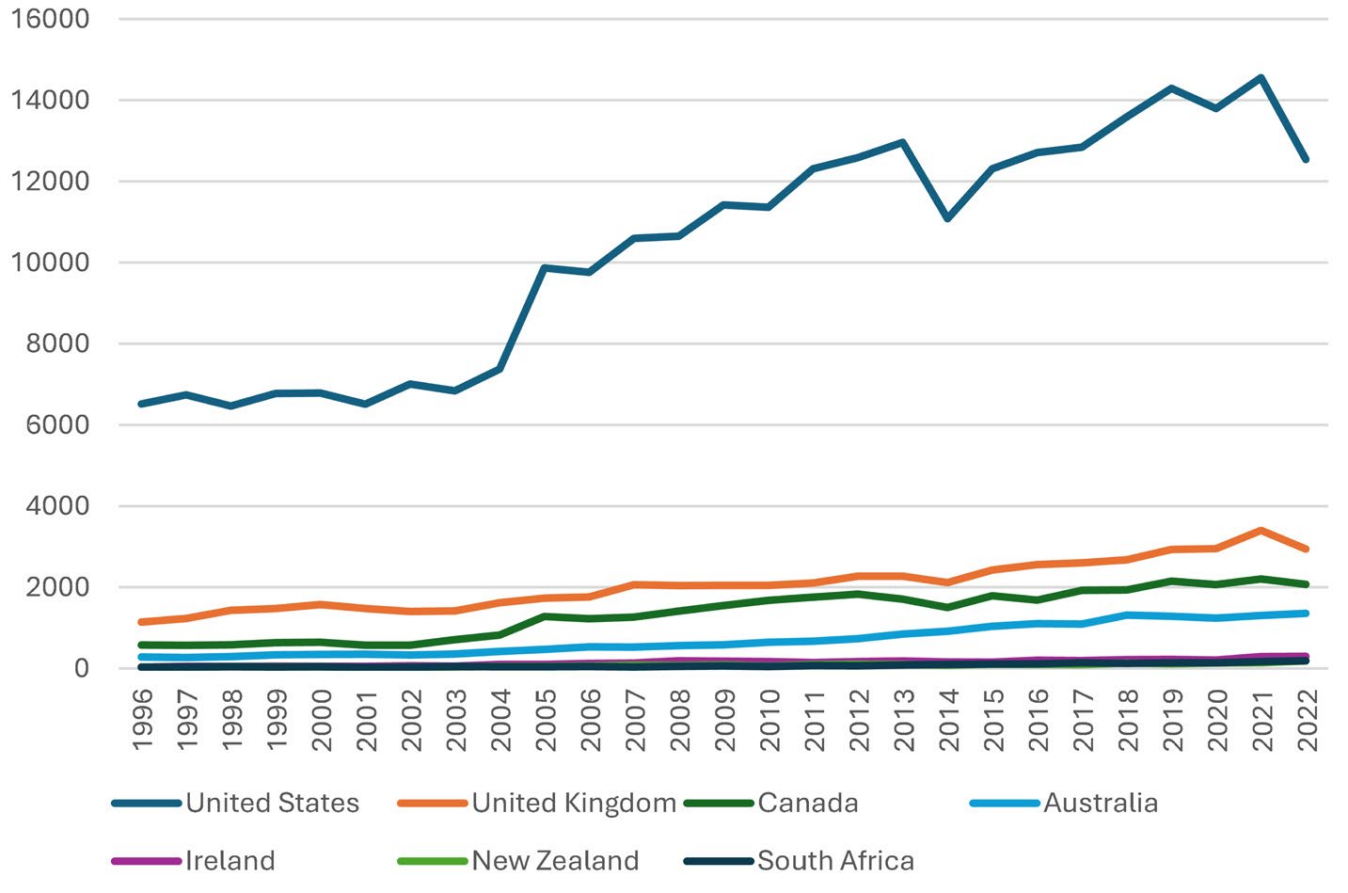
Annual RNMI publication outputs and growth trends of the selected countries between 1996 and 2022 (SJCR) [28].

All countries grew in the number of publications over the study's selected period (Figure 3), with the U.S. having a substantially greater publication output increase and maintaining its lead position over the entire period. Ireland, New Zealand, and South Africa had similar outputs with steady proportional increases over the years, whereas Canada, the U.K., and Australia showed reasonable increases in their RNMI research output. The countries largely remained in the same productivity rankings throughout the entire period, with occasional fluctuations in positions between New Zealand and South Africa.

### 3.3 Publications per Million Residents

**FIGURE 4:**
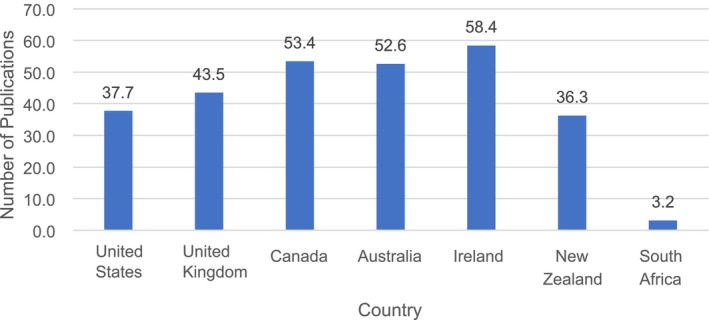
Number of RNMI publications in 2022 per million residents (SJCR) [28].

To factor in New Zealand's smaller population size, population data was extracted to examine the relative productivity of each country [21]. Factoring in each country's population size, the most productive country out of those selected was Ireland (58.4 publications per million residents), followed by Canada, Australia, and the U.K. (Figure 4). The U.S. dropped to fifth place with 37.7 publications per million residents, closely followed by New Zealand with 36.3 publications per million residents. South Africa had a notably lower number of publications (3.2 per million residents) for its population size. New Zealand and Ireland both had a population size of 5.1 million in 2022, but New Zealand exhibited notably fewer publications [21].

### 3.4 Publications per Hundred Registered Professionals

When comparing the publications per hundred registered professionals, Canada ranked first with 19.4 publications per hundred registered RNMI professionals (10669 in total). The remaining countries had notably lower publications, with Ireland ranking second (9.1; 3257), the U.K. third (7.8; 37857), and Australia fourth (7.3; 37857). New Zealand ranked fifth (5.5; 3353), and the U.S. sixth (3.6; 344424), with South Africa placing last (1.9; 10240). There was a statistically significant strong positive correlation between the number of registered RNMI professionals and the number of RNMI publications in 2022 (*r* = 0.86, *p* = 0.014).

Total citations, citable documents, self‐citations, citations per document, percentage of documents cited. When evaluating the total number of citations, citable documents and self‐citations, the U.S. remained in the lead, with 6232018 citations, 253448 citable documents and 2790144 self‐citations, followed by the U.K., Canada, Australia, and Ireland. New Zealand ranked sixth for total citations (36817), total citable documents (1967), and seventh for self‐citations (3666), while South Africa ranked seventh for total citations (33693), citable documents (1950) and sixth for self‐citations (3846) (Table 2). The U.K. ranked first for citations per document (24.12), followed by Canada, the U.S., Australia, Ireland, New Zealand (17.35), and South Africa. Interestingly, New Zealand had the greatest percentage of RNMI documents cited (77.51%), followed by the U.K., Australia, Ireland, the U.S., Canada, and South Africa.

**TABLE 2:** Total number of citations, citable documents, self‐citations, citations per document and percentage of documents cited for RNMI documents between 1996 and 2022 [27, 28].

Country	Total citations	Total citable documents	Self‐citations	Citations per document	Percentage documents cited
United States	6,232,018	253,448	2,790,144	22.24	67.43
United Kingdom	1,343,909	49,530	259,868	24.12	75.16
Canada	848,896	34,312	133,927	23.11	66.43
Australia	354,049	17,609	54,272	18.43	73.27
Ireland	67,579	3525	5161	17.53	70.71
New Zealand	36,817	1967	3666	17.35	77.51
South Africa	33,693	1950	3846	16.25	63.34

### 3.5 Category Normalised Citation Impact (CNCI), Impact Relative to World, H‐Index

To compensate for the influences of publication year, research field, and document type when undertaking citation analysis, the CNCI for each country was examined and is summarised in Table 3. It was revealed that the U.K. had the highest impact with a CNCI of 1.33, followed by Canada, the U.S., Australia, and New Zealand (1.08). These countries performed above the world average (1), while Ireland, with a CNCI of 0.94, and South Africa, with a score of 0.72, performed below the world average.

**TABLE 3:** Category Normalised Citation Impact (CNCI), citation impact relative to the world average, H‐index, and collaborations (international and industry) for each selected country's RNMI publications between 1996 and 2022 [27, 28].

Country	CNCI	Impact relative to world	H‐index	International collaborations	Industry collaborations
United States	1.22	1.30	493	71,142	11,313
United Kingdom	1.33	1.70	311	24,692	2500
Canada	1.28	1.29	265	17,417	1387
Australia	1.18	1.17	185	8557	530
Ireland	0.94	0.98	107	1914	57
New Zealand	1.08	1.01	84	1207	40
South Africa	0.72	0.64	76	1028	82

Table 3 also summarises the impact of each country's RNMI publications between 1996 and 2022 relative to the impact of global research. The U.K. had the greatest impact relative to the world average (1.70), followed by the U.S., Canada, Australia, and New Zealand (1.01). These five countries performed above the world average, whereas Ireland (0.98) and South Africa (0.64) performed below the world average. The H‐index for each country's set of RNMI publications between 1996 and 2022 is also demonstrated, where the U.S. had the highest h‐index of 493, followed by the U.K., Canada, Australia, and Ireland. New Zealand ranked second to last with an h‐index of 84, whereas South Africa had the lowest h‐index of 76.

### 3.6 International and Industry Collaborations

The extent of collaboration on RNMI publications between international co‐authors for each country from 1996 to 2022 is included in Table 3. The U.S. outperformed the other countries with 71142 international collaborations, followed by the U.K., Canada, Australia, and Ireland. New Zealand had 1207 international collaborations, closely followed by South Africa with 1028 (Table 3). The U.S. placed first with 11313 industry collaborations, followed by the U.K., Canada, and Australia. South Africa had 82 industry collaborations, exceeding that of Ireland (57) and New Zealand, which ranked last with 40 industry collaborations.

## 4 Discussion

Health research is pivotal for enhancing global health outcomes, health equity, and economic progress [6, 7]. This study examined New Zealand's RNMI research output relative to global trends using a bibliometric‐based approach analysing key performance indicators from the SJCR and InCites platforms.

### 4.1 Key Productivity and Impact Findings

The results highlighted that all seven countries, including New Zealand, had a growth in RNMI publications between 1996 and 2022, aligning with previous studies [9, 12]. This growth can be attributed to the advent of artificial intelligence and technological advancements in computing technology [12] and economic progress [29]. The U.S. retained its lead position in the total number of RNMI publications throughout the entire period and excelled in numerous categories, including the total number of citations, citable documents, self‐citations, h‐index, international collaborations, and industry collaborations. This dominance can be attributed to various factors, such as the extensive size of the American scientific community, considerable funding by American agencies, and the tendency to reference domestic journals [9].

The remaining country rankings largely remained the same across these categories, with the U.K. placing second, followed by Canada and Australia. This was followed by Ireland, whose performance often exceeded New Zealand's despite having a similar population size. New Zealand and South Africa varied in sixth and seventh places for these parameters. This order was expected since the U.S., U.K., Canada and Australia are powerhouses in the RNMI field [11, 12, 13] with greater socio‐economic development and larger population sizes [13].

To better understand New Zealand's relative research performance and ascertain whether the underperformance was due to its smaller population size and fewer registered professionals, the number of RNMI publications per million residents and per hundred registered professionals was estimated. The results found that New Zealand still underperformed in its RNMI research output, ranking sixth (36.3 publications per million residents) and fifth (5.5 publications per hundred registered professionals), indicating considerable room for improvement. Interestingly, Ireland had the highest number of publications per million residents (58.4 publications) and the second highest number of publications per hundred registered professionals (9.1 publications), while Canada led in the latter with 19.4 publications. These results suggest high productivity and a strong emphasis on research within these RNMI communities. Although Ireland and New Zealand share similar population sizes and have comparable numbers of registered professionals, Ireland outperforms in many parameters due to its unwavering commitment to engaging in and responding to evidence‐informed research, as reflected in CORU's strategic priorities for 2022–2026 [30]. New Zealand may benefit from having a similar focus placed on prioritising research endeavours.

The country rankings were noticeably different for the number of citations per document and the percentage of documents cited. The U.S. dropped to third and fifth places, respectively, whereas the U.K. ranked first in the number of citations per document. Despite New Zealand's comparatively limited productivity, New Zealand placed first in the percentage of documents cited (77.51%), which is promising as it indicates its research is widely integrated and impactful to the RNMI field. New Zealand ranked fifth for both the CNCI and Impact Relative to World, ahead of Ireland and South Africa. Not only did New Zealand outperform these countries, but it is encouraging to note that the country also marginally surpassed the world average in both categories, which incorporates a much more extensive range of countries beyond the seven specifically selected for this study.

### 4.2 Key Collaboration Findings and Implications for New Zealand

For the collaborative indicators, New Zealand was placed sixth for international collaborations and seventh for industry collaborations. Engaging in global research yields more widely applicable results, reducing geographical and demographic biases [31], whereas interdisciplinary collaboration allows for knowledge exchange and enhances the uptake of evidence‐based practices [32]. These shortcomings in New Zealand's RNMI research output can be attributed to a weak research culture, including having a low number of doctoral qualifications and the absence of clear research strategies, a professional research committee, and the knowledge and skills to conduct research [33, 34, 35]. Despite the MRTB Competence Standards [36] emphasising evidence‐based practices and understanding research methods, research is not prioritised in the strategic directions for 2020–2025 [37], nor is it a focal point in the 3‐year plan for 2023–2026 [38].

However, the recent introduction of a bachelor's degree with honours in Medical Imaging at The University of Auckland, which emphasises research [39], along with the increasing annual practising certificates [40], suggests potential growth in RNMI professionals and research output in New Zealand. This is supported by the Spearman's rank correlation coefficient, where there is a significant and positive correlation between research productivity (number of RNMI publications) and the number of registered RNMI professionals.

### 4.3 RNMI Research in Developing Countries

South Africa ranked last for every performance parameter apart from the total number of publications on the WoSCC (Figure 3), self‐citations, and industry collaborations. Medical imaging is notably underrepresented in clinical research in developing countries [41]. Although health research has fostered significant advancements in technology and healthcare in developed countries, developing countries have benefited little due to factors like limited funding and resources, lack of research culture, and difficulties accessing health information [42].

## 5 Limitations

It is important to acknowledge the limitations of this study, particularly concerning the accuracy and reliability of the data gathered.

- The choice of databases influenced the availability of publications and analysis of results. The data itself may contain inaccuracies arising from data entry errors, incomplete records, and discrepancies in citation counts. Despite these challenges, the SJCR and InCites databases are widely recognised for providing accurate publication data, and combining their data broadened the study's scope and diversity.
- Given the interdisciplinary nature of the RNMI field, the extracted data does not exclusively cover medical imaging technologists, and publications from related professions like radiologists and medical physicists would have been included.
- The study also faced challenges in obtaining accurate RNMI registration data, which varied from 2021 to 2023 based on data availability and affected the estimation of publications per registered professional, as the SJCR data was collected from the year 2022.
- Some registration data included non‐practising personnel and multiple certificate holders, with variations in the exact disciplines included in each association. The registration data was collected from publicly available sources like the annual reports from each country's professional credentialing associations, but this data was not independently verified.

## 6 Recommendations

Several important recommendations emerge when considering the future trajectory of RNMI research in New Zealand.

- Conducting a further in‐depth statistical analysis to compare New Zealand's RNMI research performance with other countries can further highlight existing gaps and provide a clear roadmap for the numbers required to align with international levels. Further research could include a detailed comparison between the number of publications and the number of registered practitioners using more accurate data obtained formally from the relevant registration bodies.
- Fostering collaborations with professional associations, local universities, hospitals, and international RNMI research leaders like the U.S., U.K., Canada and Australia can enhance the breadth and depth of future RNMI research in New Zealand. These partnerships will allow researchers to tap into a wide array of resources and expertise, boosting research productivity and strengthening the scientific impact of its publications.
- Furthermore, emphasising research in the undergraduate curriculum to develop critical writing skills and an understanding of research methodologies is essential in preparing students for future research endeavours. Moreover, securing more funds is crucial in assisting with research initiatives and will allow for more extensive studies. Establishing a dedicated research committee within the NZIMRT would also serve as a catalyst for fostering a stronger research culture within the country, potentially yielding a similar effect as witnessed in Ireland and Australia.

## 7 Conclusion

This study addresses a gap in the existing literature by examining New Zealand's RNMI research output compared to selected countries. Through using a bibliometric approach and analysing key performance parameters from the SJCR and InCites platforms, it was found that while the trajectory of RNMI publications in New Zealand is following the global upward trend, it does so at a proportionate loss, despite the country's smaller population size and lower number of registered RNMI professionals. However, the study did reveal that New Zealand's RNMI publications are relevant and high quality, as evidenced by their notable citation impact.

This study adds depth to the understanding of RNMI research in New Zealand and also serves as a valuable resource for researchers, health professionals, and policymakers internationally. Nevertheless, there is room for improvement in enhancing the productivity and scientific impact of New Zealand's RNMI research. Undertaking purposeful initiatives and collaborative endeavours to yield tangible and impactful outcomes for the RNMI research community of New Zealand will pave the way for improved health outcomes for the future.

## 8 Conflicts of Interest

The authors declare no conflicts of interest.

## Data Availability

The data that support the findings of this study are openly available in https://incites.clarivate.com.
